# Current Role of Monoclonal Antibody Therapy in Pediatric IBD: A Special Focus on Therapeutic Drug Monitoring and Treat-to-Target Strategies

**DOI:** 10.3390/children10040634

**Published:** 2023-03-28

**Authors:** Merle Claßen, André Hoerning

**Affiliations:** 1 Department of Pediatrics and Adolescent Medicine, University Hospital Erlangen, Friedrich-Alexander-University-Erlangen-Nuremberg, 91054 Erlangen, Germany; 2 German Center Immunotherapy, DZI, Ulmenweg 18, 91054 Erlangen, Germany

**Keywords:** Crohn’s disease, ulcerative colitis, biologics, treatment optimization, trough levels

## Abstract

In the last two decades, biologicals have become essential in treating children and adolescents with inflammatory bowel disease. TNF-α inhibitors (infliximab, adalimumab and golimumab) are preferentially used. Recent studies suggest that early application of TNF-α inhibitors is beneficial to inducing disease remission and preventing complications such as development of penetrating ulcers and fistulas. However, treatment failure occurs in about one third of pediatric patients. Particularly, children and adolescents differ in drug clearance, emphasizing the importance of pharmacokinetic drug monitoring in the pediatric setting. Here, current data on the choice and effectiveness of biologicals and therapeutic drug monitoring strategies are reviewed.

## 1. Introduction

The use of biologicals in pediatric patients with inflammatory bowel disease (IBD) has widely increased since their introduction [1]. Biologicals have substantially improved the disease course for many pediatric patients suffering from Crohn’s disease (CD) and ulcerative colitis (UC). Current guidelines recommend the use of TNF-α inhibitors in pediatric IBD patients with high disease activity or in those who do not respond to other therapeutic strategies [2,3]. Infliximab and adalimumab were approved for the treatment of pediatric Crohn’s disease in 2006 and 2012, respectively, and later for pediatric UC also. These compounds are thus the most recommended. As a result, the data on the use of biologics in pediatric and adolescent patients with IBD have increased in number significantly in recent years. Biologic agents are effective and safe, and they are one of the most used medication classes in pediatric IBD [4].

**Figure 1:**
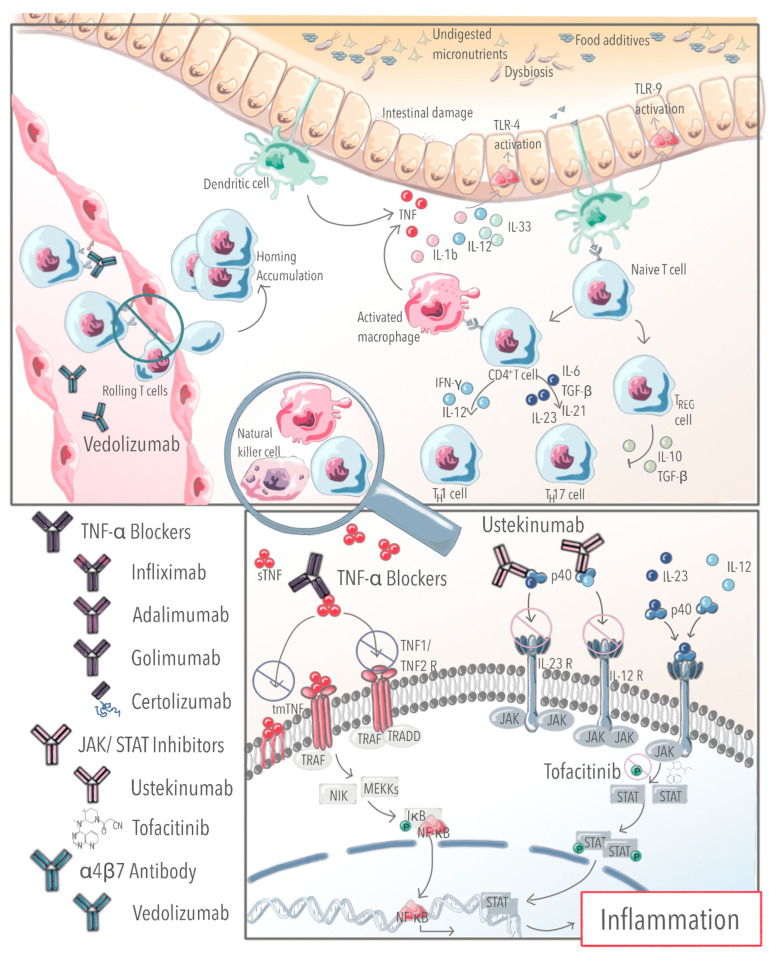
Overview of pathophysiological mechanisms and the effects of biologic agents currently used in pediatric IBD.

In a large German cohort (CEDATA), adalimumab was the most commonly used biologic when therapy with infliximab failed. According to Cozijnsen and colleagues, this approach was effective in a small retrospective study [5]. Infliximab, adalimumab and golimumab bind sTNF and mTNF, while etanercept only binds soluble TNFu [6]. The reason for the use of adalimumab after infliximab treatment failure may be that other biological agents lack official approval in childhood and are used off-label despite being available for treatment in adults. However, information on treatment success exists in children for alternative biologicals such as golimumab, certolizumab (both TNF-α inhibitors), vedolizumab, an α4β7-integrin blocker, and ustekinumab, an IL-12/IL-23 blocker (see Figure 1 for an overview of currently used biologicals in pediatric IBD). Approximately 54% of 42 pediatric IBD patients treated with vedolizumab entered clinical remission within 14 weeks, even when prior treatment with anti-TNF-α drugs had failed, according to a retrospective multicenter study [7]. Another large multicenter study demonstrated ustekinumab to be safe and effective in the treatment of Crohn’s disease [8]. Pediatric patients with UC achieved deep mucosal remission when treated with ustekinumab, even when a relapse occurred under therapy with infliximab and vedolizumab [9]. Similarly, golimumab resulted in clinical remission [10]. Ninety percent of TNF-α naïve patients remained steroid-free compared to 50% of patients who did not respond to other biologic therapies [10]. Therefore, European guidelines recommend treatment with infliximab or adalimumab as an effective option for pediatric patients with moderate-to-severe Crohn’s disease. After the failure of anti-TNF-α or other treatments, ustekinumab and vedolizumab are recommended [2,3].

## 2. Clinical Effect of Biologics

The large German–Austrian registry study of GPGE (CEDATA) showed that pediatric patients with high disease activity were significantly more likely to receive biologic agents, preferably infliximab [1]. Treatment is most likely to last approximately one year, but only because the surveillance ended [1,11]. The discontinuation rate was 3.2% per year due to a loss of response in a Canadian study [11]. Treatment outcomes suggest that patients with high disease activity in Crohn’s disease respond equally well to infliximab and adalimumab, although randomized controlled head-to-head studies are lacking [12,13]. After three months of treatment, infliximab induced significant mucosal healing and was associated with improvement in clinical disease scores in adults [14]. Similarly, patients with Crohn’s disease treated with adalimumab experienced mucosal healing [15]. Scarallo and colleagues also found that infliximab and adalimumab induced mucosal and histologic healing in about 40% of pediatric patients with CD and UC [16]. Inflammatory markers such as C-reactive protein (CRP) and white blood cell count decreased in children and adolescents with moderate-to-severe Crohn’s disease on TNF-α inhibitor therapy [17]. Compared with enteral nutrition therapy alone, biological therapy is similarly effective in inducing mucosal remission in patients with Crohn’s disease and significantly improves the quality of life [18,19]. A review of clinical trials demonstrated the long-term therapeutic benefit of infliximab in pediatric patients with Crohn’s disease on continuous therapy [20]. With anti-TNF-α therapy, approximately 60% of pediatric patients with perianal CD respond well to treatment, and 40% achieve sustained remission [21].

In moderate-to-severe ulcerative colitis, treatment with infliximab led to remission and was safe [22]. The treatment with infliximab in pediatric patients with ulcerative colitis is associated with a lower frequency of colectomy compared with other treatment options [23]. Adalimumab also showed good results in the double-blind ENVISION I trial to treat children with moderate-to-severe ulcerative colitis, with a higher induction dosage yielding better results [24]. However, treatment with biologics did not affect the number of hospitalizations in general [23]. Comparing the years before the introduction of biologics in children and adolescents with Crohn’s disease, the time thereafter showed less disease progression to stenosing disease and fewer surgeries but unaltered overall hospitalizations [25].

Pediatric patients with IBD also gained weight and, especially, grew up to the same height as healthy controls when treated with TNF-α blockers. This also led to a significant increase in physical activity, while the overall health-related quality of life remained unchanged when compared to pre-anti-TNF-α treatment [26].

## 3. Methods and Selection Criteria

A non-systematic literature search of PubMed was performed in January 2023, using the following search terms: (“Crohn Disease”[Mesh] OR “Inflammatory Bowel Diseases”[Mesh] OR “Colitis, Ulcerative”[Mesh] OR “Pediatric ulcerative colitis” [Supplementary Concept] OR “Pediatric Crohn’s disease” [Supplementary Concept]) AND (“Infliximab”[Mesh] OR “Adalimumab”[Mesh] OR “Tumor Necrosis Factor Inhibitors” [Pharmacological Action] OR “vedolizumab” [Supplementary Concept] OR “Ustekinumab”[Mesh] OR “golimumab” [Supplementary Concept] OR “tofacitinib” [Supplementary Concept]) AND (“Child”[Mesh] OR “Adolescent”[Mesh]). Additionally, the references of selected studies were screened for further studies. The inclusion criteria were a pediatric sample with IBD and the use of a biological agent such as infliximab, adalimumab, golimumab, ustekinumab and vedolizumab. The main aim was to include current literature, so mostly studies of retrospective or prospective nature, meta-analysis, and case reports since 2020 were considered, but we included earlier studies and adult studies if evidence was lacking (see Table 1 for all included pediatric studies).

## 4. Early and Effective Use of TNF-α Blockers Prevents Disease Progression and Disease Complications

Evidence suggests that early treatment with biological agents is favorable for pediatric CD patients [1,28,29]. Early application of biological agents significantly prevented treatment failure [1]. In 2020, the first randomized direct comparison of first-line infliximab with exclusive enteral nutrition or corticosteroids as first-line treatment in pediatric patients with moderate-to-severe Crohn’s disease was provided by Jongsma and colleagues [50]. Of the patients treated with first-line anti-TNF-α therapy, a significantly higher percentage accomplished clinical and endoscopic remission [50]. In addition, first-line TNF-α-blocker therapy needed less dose escalation while achieving mucosal healing [50]. Consistent with this, data from the CEDATA registry study showed that first-line infliximab therapy led to a higher rate of clinical remission in the short term compared to conventional therapy with biologics, which led to endoscopic remission in another study [1,50]. In addition, Jongsma and colleagues found that the probability of continued clinical remission at week 52 with monotherapy of azathioprine was higher in children who received infliximab as first-line therapy to induce remission [50]. Comparable results were shown for adalimumab [72]. In Crohn’s disease, early and effective use of TNF-α blockers also prevented the development of disease complications, for example, strictures or penetrating ulcerations and disease progression [28]. A Korean study revealed that early anti-TNF-α medication led to a lower risk of surgery during disease progression [30]. In another study, early admission of biologics significantly reduced the risk of penetrating complications but not stenosing complications [31].

A large cohort study showed that unrelated to the IBD subtype, the administration of biologicals a short time after diagnosis (<120 days) is connected to fewer glucocorticoids being needed [32]. Another large cohort study of pediatric patients with IBD demonstrated that the early treatment with TNF-α blockers was superior to immunomodulators in achieving remission within three months [29]. In a retrospective Canadian study, earlier initiation of anti-TNF-α treatment in patients with Crohn’s disease and ulcerative colitis was more common in adolescents and was associated with higher PCDAI/PUCAI and lower serum albumin levels at diagnosis [33].

The incidence of extraintestinal manifestation in IBD throughout treatment with biologicals ranges from 14% to 25% [1,78]. However, the study with the longer follow-up reported a higher incidence of extraintestinal manifestations [1]. In the large German multicenter cohort, first-line prescription significantly reduced the incidence of extraintestinal manifestations longitudinally [1]. These patients had the highest disease activity due to systemic inflammation before treatment, so the findings are promising. Moreover, the incidence of extraintestinal manifestations was reduced from about 27% to 25% immediately after treatment initiation, with a further reduction to 17% at six months [1].

## 5. Occurrence and Frequency of Adverse Treatment Events

In several studies evaluating different biologicals, adverse treatment events occurred in around 46% of patients within all IBD subtypes [1,79]. Immediate infusion reactions (11%) and a psoriasis-like rash (11%) were reported [34,79]. Symptoms of infusion reactions include dyspnea, coughing, cyanosis and vomiting [19]. Minor infections were reported in 15.4% of patients [34].

Several studies have reported varying rates of skin complications due to biological treatment ranging from 13% to 39%, with the most recent study reporting 17% [1,36,37]. Even for golimumab, severe skin reactions were the reason for discontinuation in a case study of adults with CD [80]. Dolinger et al. recommend switching to ustekinumab in the event of skin reactions on TNF-α inhibitor therapy. In the study by Nuti et al., a psoriasis-like rash was observed in 11% of patients treated with infliximab or adalimumab [34]. The risk of skin adverse events appears to be increased only with adalimumab and not with infliximab in patients with IBD and juvenile idiopathic arthritis [38]. There appears to be no association between higher drug concentrations and increased adverse event rates [39]. Vedolizumab and ustekinumab showed a good safety profile [79,81]. The most common adverse events were respiratory tract infections (33%) with vedolizumab [79] and infusion reactions with ustekinumab [10]. Additional adverse events related to vedolizumab were headaches (4%) and myalgia (3%), while only 1% of patients discontinued treatment due to adverse events in a multicenter cohort study [75]. More serious adverse events are rarely observed and are described in case reports. The currently available evidence suggests that treatment with TNF-α inhibitors is associated with a very low risk of developing malignancies. These almost exclusively occurred when combining TNF-α blockers with azathioprine in male patients [35].

## 6. Therapeutic Drug Monitoring to Optimize the Treatment Strategy and Maintain the Efficacy of Biological Agents

Primary and secondary treatment failures with anti-TNF-α drugs are common and challenging in daily clinical practice. Approximately 10% to 30% of adult patients experience primary non-response [82], while 20% to 50% develop secondary loss of response during biological therapy [82]. A large retrospective registry study of adults with ulcerative colitis showed that around 50% of the patients had a suboptimal response to anti-TNF-α agents, leading to dose escalation or treatment discontinuation [83]. The main causes of primary non-response or loss of response are low trough levels or anti-drug antibodies, respectively [2]. For preventing treatment failure and also for following a treat-to-target strategy, optimal dosing is important for achieving not only clinical remission but also mucosal healing as one of the most important long-term goals [84]. Short-term goals that reflect adequate therapy management include the normalization of inflammation markers in the serum and feces [84].

A meta-analysis by the ESPGHAN-IBD working group suggests that a higher dose per kilogram of body weight may be appropriate in younger IBD patients, as they often have lower trough levels in the early phase of therapy induction [51]. Indeed, a retrospective British study demonstrated that children with very early onset IBD received the increased dose of 10 mg/kg body weight [40]. The remission rate in these patients was 62%; otherwise, the course of very young children with IBD treated with biologics appears to be similar to that of older patients [40,41]. In particular, studies in pediatric patients with ulcerative colitis and Crohn’s disease have shown that higher infliximab trough levels after induction predict remission one year after infliximab administration [52,53] (see also Table 2).

A large UK prospective observational study (PANTS) of approximately 1000 children and adults with IBD tried to identify clinical and pharmacokinetic factors that might predict primary non-response at week 14, non-remission at week 54, and adverse events leading to drug discontinuation [42]. In the multivariable regression analysis, the only factor independently associated with a primary non-response was low drug trough levels of infliximab and adalimumab at week 14 [42]. Approximately 63% of the patients developed anti-drug antibodies to infliximab and 29% to adalimumab [42]. For both drugs, suboptimal drug concentrations at week 14 predicted immunogenicity and the development of neutralizing anti-drug antibodies predicted subsequent low drug concentrations [42]. A further important finding was that a combination immunomodulatory therapy (thiopurine or methotrexate) reduced the risk of anti-drug antibody development for infliximab and adalimumab [42].

Therapeutic drug monitoring (TDM) is advocated to assess trough levels and/or neutralizing anti-drug antibodies (ADA) to optimize the treatment strategy and maintain the efficacy of biological agents. TDM can either be reactive or proactive. In reactive TDM, drug concentrations and/or the occurrence of ADAs are assessed in the serum in case of persistent or recurrent flares of IBD. Reactive TDM can streamline the management of primary non-response and secondary loss of response. If the drug concentration appears to be subtherapeutic, the dose may either be increased or the interval between the doses reduced. If the ADA titer is low, adding an immunomodulator to the biologic treatment should be considered. In case of a high ADA titer, the biological agent may be switched to another biologic in the same class or a different class.

Proactive TDM signifies the assessment of drugs’ trough levels during remission to ensure effective therapy, prevent a disease relapse by maintaining adequate drug levels, and reduce the formation of ADA. Proactive TDM increases clinical remission and the durability of the response to a biological agent [69]. In recent years, guidelines and consensus statements have been published on the emerging topic of TDM for adults [85,86,87,88,89,90] and children [2,3]. These guidelines recommend that reactive TDM to guide treatment in patients with biologicals is more cost-effective than empiric dose escalation.

In recent years, randomized control trials such as the Pediatric Crohn’s Disease Adalimumab Level-based Optimization Treatment (PAILOT) trial [69] and the NOR-DRUM B study have suggested the utility of proactive TDM [91]. Proactive drug monitoring of adalimumab in the randomized PAILOT trial was associated with significantly higher rates of corticoid-free remission and lower inflammatory markers [69]. Infliximab trough levels greater than 10 mg/mL are generally associated with remission and higher rates of perianal fistula healing in pediatric IBD patients [54,92]. Yarur and colleagues recommend in adults a treat-to-target strategy until adequate infliximab levels are achieved [92]. Of note, current data indicate that an infliximab or adalimumab therapy should generally not be discontinued unless drug levels are greater than 10 μg/mL [92].

As higher infliximab levels after induction were associated with clinical remission [55], proactive drug monitoring in the induction phase of infliximab was associated with optimal trough levels at week 52 and clinical remission in pediatric IBD patients [56]. In particular, early response and drug monitoring during induction appear to predict response rates, possibly due to higher drug clearance in children and an association with higher cytokine levels at diagnosis [57,58]. Higher drug clearance was associated with hypoalbuminemia, high CRP, higher BMI, male sex and anti-drug antibodies [59,81,93]. A small Spanish study points out that proactive drug monitoring during maintenance is favorable in order to maintain long term clinical response and showed response rates of 92.8% after three years in pediatric patients with Crohn’s disease [43].

In addition, in adults, a high initial serum TNF-α and a severe inflammation with extensive mucosal involvement leads to increased drug consumption [94] and fecal loss [95], while younger age (<10 years) is attributed to different pharmacokinetics in children compared to adults [51,96]. This has led to the revised recommendation of an intensified infliximab treatment (10 mg/kg body weight at weeks 0, 1 and 4) to achieve remission in cases of an acute severe colitis by the ESPGHAN in 2018 [97].

In a small Spanish cohort of pediatric patients with Crohn’s disease, proactive drug monitoring (measurement of trough levels) prevented loss of response to infliximab and adalimumab due to antibodies [43]. Anti-drug antibodies are associated with loss of response to infliximab [60]. In patients who have already developed anti-drug antibodies, dose escalation of the biological drug suppressed anti-drug antibodies in the subsequent study [43,45]. Another approach to suppress anti-drug antibodies is to combine the biologic with an immunomodulator which is supported by the evidence for infliximab [46] but not for adalimumab [70] in pediatric patients with Crohn’s disease. Patients receiving infliximab as a second-line treatment for failed therapy benefit significantly from combination therapy with immunomodulators [20]. In general, combination therapy increased the likelihood of continuing infliximab at two years [20].

For several of the above-mentioned reasons, the dose of the biological drug does not necessarily correspond to the determined drug trough levels [54]. Therefore, a Bayesian calculation model applied to drug concentrations represents a new approach to optimize treatment response to biologics in IBD by incorporating several individual parameters that affect drug clearance, such as sex, hypoalbuminemia, and fecal loss [54]. It predicts the treatment response to optimize dosing [98]. With the implementation of three trough-level measurements, the model was able to predict drug concentrations and thus be helpful for therapy adjustments [54]. Precision dosing showed better remission and response rates in adults compared to traditional dosing regimens [61]. Sufficient models for children in clinical practice have yet to be determined due to the large amount of data needed to test the robustness and identify an appropriate computational model to predict individual drug concentrations. However, data on TDM in pediatric IBD are emerging and allow for recommendations for treatment monitoring (summarized in Table 2). Notably, to date, the superiority of proactive TDM has not been consistently demonstrated in randomized controlled trials [99].

**Table 2:** Target trough levels during induction and maintenance as reported from recent studies.

	Induction	Maintenance
Infliximab	>18 µg/mL before 3rd infusion to achieve clinical remission in CD [63] >12.7 µg/mL before 4th infusion for fistula healing and >9.1 to prevent treatment failure in CD [64,65] >13 ug/mL before 4th infusion for fistula healing (*) [66]	>7µg/mL to prevent treatment failure in CD [42]—>8.3 µg/mL for clinical remission in CD [67] >10.1 µg/mL for fistula healing in CD [92]
Adalimumab	>13.85 µg/mL at the end of induction for long term clinical remission in UC and CD [73]	≥10.1 μg/mL–12 µg/mL (*) to prevent treatment failure [42] In case of loss of response—>new induction dose or weekly application [71,100]
Golimumab		>0.97 μg/mL at week 14 for clinical response in UC [101]
Ustekinumab		≈6.6 µg/mL at week 8 (associated with steroid-free remission week 52) in all IBD subtypes [10]
Vedolizumab	>37 µg/mL before 3rd infusion and >20 µg/mL before 4th infusion to achieve steroid free-clinical remission in UC and CD [77] >30 µg/mL in week 2 (*) for endoscopic remission, clinical remission in CD and UC [102]	<30 kg: >7 μg/mL for steroid free and EEN-free remission in all IBD subtypes [75] >30 kg: ≥11.5 µg/mL for clinical and biochemical remission in CD and UC (*) [76]

Note that not all studies performed cut-off tests for trough levels and some studies did not find an association between trough levels and disease outcome (e.g., [9,103]), (*) = adult studies.

Surgical interventions and partial bowel resection for Crohn’s disease still represent a rescue option. However, compared to the beginning of the 2000’s, these procedures are less frequent, especially in patients responding to TNF-α inhibitors [68]. It is well known, especially in pediatric Crohn’s disease patients, that the postoperative recurrence risk after surgery is substantial. In a pediatric series, clinical recurrence rates after partial intestinal resection were 17% at 1 year, 38% at 3 years and 60% at 5 years [104]. Therefore, a postoperative remission-maintaining therapy should be used after surgically induced remission in children, as recommended by the ECCO/ESPGHAN expert committee [2,105]. Thiopurine is recommended as the first choice for postoperative relapse prophylaxis in IFX-naïve patients and anti-TNF-α antibodies in high-risk cases. While in pediatric IBD, randomized controlled trials on this topic are lacking, supporting data for the postoperative use of anti-TNF-α therapy to reduce the risk of recurrence at the anastomoses was reported by three RCTs conducted in adult patients with ileocolonic resections and primary anastomoses [106,107,108]. A recent German study reported a reduced endoscopic recurrence after ileocecal resection in children receiving preoperative TNF-α inhibitors [49].

In summary, the quality and efficacy of treatment in pediatric IBD appear to have improved, as children with Crohn’s disease suffer fewer relapses in the last five years than 10–15 years ago [47]. Early treatment with infliximab or adalimumab should be considered if patients are at high risk of a poor outcome, e.g., Crohn’s with persistently high disease activity despite adequate induction therapy, extensive or pan-enteric manifestation, deep colonic ulcerations, marked growth retardation, severe perianal involvement, radiologically or endoscopically proven structures, the occurrence of fistulas, intestinal perforations, inflammatory conglomerates and/or abscesses, and CMV infections [2]. Similar features in ulcerative colitis qualify for a TNF-α inhibitor as pancolitis, extensive and deep colonic ulcerations, the early need for (recurrent) steroid therapy, and recurrent infections with Clostridioides difficile or CMV [3,97].

Additional recommendations will soon further refine biological therapy strategies; for example, trials are comparing longer dosing intervals in children in remission on TNF-α blockers [109], which would further improve the quality of life of children. One study even showed that discontinuation of biologics could be considered if endoscopic and histologic remission occurs in children with ulcerative colitis on TNF-α blockers [48]. Individualized medicine, considering pharmacogenetic and pharmacogenomic aspects, is expected to lead to further advances in treatment. For example, a study of response to infliximab found that a variant in the FCGR3A gene was associated with a decreased response to infliximab with lower levels and higher anti-IFX antibody concentrations [62]. HLA polymorphisms (G allele of rs2395185 and the C allele of rs2097432) were associated with reduced long-term response in adults but not children with CD to anti-TNF-α medication [44]. So pharmacological models might have to take different polymorphisms in children and adults into account.

## 7. Conclusions

TNF-α blockers are a safe and efficient way to treat IBD with high disease activity in children and adolescents. Infliximab and adalimumab are efficient in achieving clinical and mucosal remission. However, as treatment failure still occurs, therapeutic drug monitoring and exclusion of the formation of anti-drug antibodies are helpful for further treatment management. For both infliximab and adalimumab, drug concentrations to achieve different treatment goals are available. Therapeutic drug monitoring involves a proactive and a reactive strategy, yet further prospective RCTs are still needed to pose recommendations for which one to prefer. For other monoclonal antibodies, such as vedolizumab and ustekinumab, favorable drug concentrations are mostly derived from adult studies.

## Figures and Tables

**Table 1:** All included studies with pediatric patients and biologics.

Study	N TNF	Age (Yrs) (CD, UC)	m % (CD, UC)	Study Type	Observation Period	Country	IBD Type	Biologic	Time from Diagnosis to Biologic	Comparison-Group	Post-Assessments	Outcomes	Results
Claßen et al., 2022 [1]	487	11.9	59.1	Retrospective registry	2004–2020	Germany, Austria, Switzerland	CD, CU, IBDu	all	19 months	First-line vs. Second line		Laboratory markers, clinical scores, side effects, treatment failure	Patients with CD significantly benefitted from early treatment, with lower clinical scores, fewer EIMs and lower risk for treatment failure
D’Arcangelo et al., 2021 [27]	185	13	58	Retrospective, observational cohort Single-center	2012–2020	Italy	CD, UC, IBDu	IFX, ADL, UST, VEDO	2 yrs			Immediate and delayed AEs	32.8% biologic-related Aes 10% immediate reactions, 45% delayed 14% treatment discontinuation because of AEs
Kaplan et al., 2023 [4]	17,649			Retrospective, observational cohort	2006–2016	USA	CD, UC	all				Use, discontinuation	43% of pediatric IBD patients treated with biologic, more likely for CD, discontinuation significantly higher in UC
**TNF-α inhibitors**													
Bronsky et al., 2022 [13]	62	11.64–16.27	55–68	Prospective observational cohort	2013–2017	Czech Republic	CD	IFX, ADL	0.6–1.04 yrs	IFX vs. ADL	Up to 24 months	Treatment escalation Non-response Serious AEs	No difference between IFX and ADL in efficacy and safety
Lee et al., 2015 [18]	52	13.9	46	Observational cohort		Canada, USA	CD	IFX (1xADL)	0.7 yr	EEN (*n* = 22), PEN (*n* = 16)	8 weeks	PCDAI, QoL, mucosal healing via FCP	Clinical response: 64% PEN, 88% EEN, 84% TNFi Mucosal healing: PEN 14%, EEN 45%, TNFi 62% QoL not statistically significant
Scarallo et al., 2021 [16]	134	10.9, 10.3	65.4, 50	retrospective, observational (two centers)	2008–2018	Italy	78 (CD) 56 (UC)	IFX, ADL				Endoscopically assessed mucosal remission	Mucosal remission in 41% of CD patients and 53.6% of UC patients, histological remission in 33.3% of CD patients and 39.3% of UC patients
Boros et al., 2023 [26]	32	15.2, 16.4	49	Prospective, observational follow up Single-center	2016–2018	Hungary	CD, UC	TNFi	1.4 yrs, 3 yrs	Healthy controls	2 & 6 months	Body composition, health-related quality of life, physical activity	Body composition and physical activity significantly improved after 6 months and caught up to healthy controls, no change in health-related quality of life 58% of CD 37.5% of UC patients in remission
Kim et al., 2021 [28]	84	15	74.1	Retrospective single-center	2000–2013	Korea	CD	TNFi		Thiopurine treatment (N = 287)	Up to 13 yrs	Disease behavior evolution	Early treatment (within 3 months after diagnosis) was associated with lower risk of disease behavior progression
Walters et al., 2014 [29]	68	11.8	61	Retrospective multicenter	2008–2012	North America	CD	IFX, ADL	Within 3 months	Immunomodulator (IM) (N = 68), no IM (N 68)		Steroid-free and surgery-free remission, growth	85.3% in remission with TNFi, significantly more than other groups, growth improved in biologic group only
Ley et al., 2022 [25]	1007			Retrospective multicenter	1988–2011	France	CD	TNFi			M = 8.8 yrs	Intestinal resection, disease progression, hospitalizations	Reduction in intestinal resection and disease progression, no change in hospitalization over time
Choe et al., 2022 [30]		Pediatric + adult		Population-based	2006–2015	Korea	CD, UC	TNFi				TNFi prescription, fistulectomy, surgery	Lower odds of surgery in CD patients under TNFi therapy
Kugathasan et al., 2017 [31]	913			Prospective inception cohort	2008–2012	USA, Canada (28 sites)	CD	TNFi				Disease complications	Early TNFi admission reduced risk for penetrating complications but not stricturing complications
Kandavel et al., 2021 [32]	27,321			Retrospective cohort, multicenter	2007–2018	US, UK, Qatar	CD, UC, IBDu	TNFi				Use of corticosteroids	Appliance of TNFi within the first 120 days after diagnosis reduces risk for need of steroids later in CD not in UC
Sherlock et al., 2021 [33]	198	10.5	59.1	Retrospective cohort, single-center	2001–2015	Canada	CD, UC, IBDu		21.5 months		M = 47.8		Biologic therapy associated with older age, higher PCDAI/ PUCAI hypoalbuminemia in UC and CD
Nuti et al., 2014 [34]	78	15	63	Single-center cohort	2001–2011	Italy	CD	IFX, ADL	40.6 months		1, 2, 3 yrs	Clinical activity (PCDAI), discontinuation, AEs	81% continuation yr 1, 54% yr 2, 33% yr 3, no serious AEs
Beukelmann et al., 2018 [35]	6808		43	Retrospective, cohort		US	IBD, JIA, PsA	TNFi		No TNFi use (N = 20,049)		Malignancies	TNFi use in combination with thiopurines increased the risk for malignancies
Hradsky et al., 2021 [36]	100	15	57–65	Retrospective			CD	TNFi				Skin complications	After 2 yrs of treatment 35% of patients developed at least one skin complication
Dolinger et al., 2022 [37]	638			Retrospective				IFX, ADL			6 months	Skin reactions	21% infliximab patients, 11% adalimumab patients
Baggett et al., 2022 [38]	3794			Retrospective	2008–2020			IFX, ADL, etanercept		Non-TNFi exposure		Incidence of psoriasis	Higher risk of psoriasis in patients treated with TNFi (highest in adalimumab)
Zvuloni et al., 2021 [39]	135	12.9	56.3	Retrospective, cohort single-center	2015–2020	Israel	CD, UC	IFX, ADL			MD = 1.7 yrs	Incidence of AEs	37% of patients had AEs, psoriatiform rashes (45%), elevated transaminases (32%) and infusion reactions (13%)
Eindor-Abarbanel et al., 2022 [40]	89	3.8	62.8	Retrospective	2005–2019	United states	VEO IBD	TNFi		TNFi-naive	1 yr	Disease course, dose, and dose interval of IFX	39.5% of VEO IBD patients received TNFi, higher disease activity was associated with TNFi-treatment, clinical remission on first biologic agent in 61,8%
Kerur et al., 2022 [41]	294			Retrospective, cohort Multicenter	2008–2013	North America	VEO IBD	IFX, ADL			1, 3, 5 yrs	Utilization and durability of TNFi	55% of patients treated with TNFi between 0–6 yrs old, durability 90% after one yr, 55% after 5 yrs, lower durability in UC und IBDu
Kennedy et al., 2019 [42]	219 pediatric	Adult + pediatric	49	Prospective, observational cohort, multicenter	2013–2016	UK, Korea, USA	CD	IFX, ADL	2.3–3.3 yrs		12 months	Disease activity, AEs, discontinuation, treatment failure, anti-drug antibodies	Low drug concentration the only predictor for primary non-response in week 14, and remission by week 54 62.8% ADAs in IFX, 28.5% in ADL predicted by suboptimal drug concentration in week 14
Rodriguez Azor et al., 2023 [43]	30	11.3	70	Prospective observational	2015–2020		CD	IFX, ADL	9.9 months		M = 27.1 months	Clinical remission, mucosal healing, laboratory markers	87.1% in clinical remission (wPCDAI), 83% achieved mucosal healing (MINI)
Salvador-Martín et al., 2023 [44]	340	11.2	60.3	Observational, multicenter		Spain	CD, UC, IBDu	IFX, ADL	6.1 months	Responders vs. non-responders	9 yrs	Treatmtent failure	Only in adults association of HLA polymorphisms and treatment failure
Cohen et al., 2019 [45]	234	13	54.2	Retrospective, single-center		USA	CD, UC	IFX, ADL		With and without ADAs		ADAs	24.8% developed ADAs, 48% of those underwent dose optimization and of those 54% had undetectable ADAs on follow-up, Patients switching to another agent were not more likely to develop ADAs
Colman et al., 2021 [46]	89	12.2–17.7	58.7	Retrospective cohort, single-center	2014–2018	USA	CD, UC, IBDu	IFX, ADL		With and without immunomodulator (IM)	6, 12 months	Clinical and biochemical remission, discontinuation, ADAs	Significantly more patients in combination therapy with TNFi and IM were in remission after one yr than without IM (53.9% vs. 26.8%) Without IM ADAs were unlikely to reverse if titer > 329 ng/ml
Sassine et al., 2022 [47]	639	14	56	Retrospective cohort study	2009–2019		lCD	TNFi				Clinical relapse	Use of TNFi reduced risk for relapse compared to immunomodulators
Scarallo et al., 2021b [48]	170	12	65.6, 46.7	Retrospective Two centers	2008–2018	Italy	CD, UC	IFX, ADL	1–1.5			Endoscopic (mucosal and histological) remission	MH was achieved by 32 patients with CD (41%) and 30 patients with UC (53.6%); 26 patients with CD (33.3%) and 22 patients with UC (39.3%) achieved HH Withdrawal of TNFi associated with relapse
Weigl et al., 2023 [49]	13		52	Retrospective		Germany	CD	TNFi		No perioperative TNFi (N = 16)		Weight, height, disease activity, infections	Improvement of weight, height after ileocecal resection, significantly more improvement in disease activity in TNFi group, no increase in infections
**Infliximab**													
deBruyn et al., 2018 [11]	180	14.3	54.4	Retrospective, multicenter	2008–2012	Canada	CD	IFX	1.5 yrs			Discontinuation, dose optimization	Dose escalation occurred in 57.3% primarily due to loss of response Annual discontinuation 3.2% per yr
Kierkus et al., 2012 [17]	66	14.1	43.9	Prospective cohort		Poland	CD	IFX	5.6 yrs		2, 6, 10 weeks	Disease activity (clinical, laboratory & endoscopic)	33% reached clinical remission, 28% non-responders, endoscopic improvement in week 10
Luo et al., 2017 [19]	13	11.7	46.2	Prospective		China	CD	IFX	12 months	EEN (*n* = 13)	8 weeks	PCDAI, growth, AEs	Significantly higher percentage of clinical response, growth, and AEs in IFX group
Hyams et al., 2012 [22]	60	14.5	53.3	Randomized	2006–2010	USA, Canada	UC	IFX	1.4 yrs	Dosing interval 8 vs. 12 weeks	8, 54 weeks	Clinical remission, AEs	Response at week 8 73.3%, overall remission rate at week 54 was 28.6%, no serious AEs
Bolia et al., 2019 [23]	204	12	50	Retrospective	2005–2016	Australia	UC	IFX				Colectomy rates	Reduction in colectomy rates after introduction of IFX
Jongsma et al., 2020 [50]	50			Multicenter open label randomized controlled trial			CD	IFX		Conventional treatment (N = 50) Steroids/EEN	10, 52 weeks	Clinical and endoscopic remission	Higher percentage of patients in TNFi group achieved clinical (59%) and endoscopic remission (59%) at week 10, no significant difference in week 52, less treatment escalation needed in TNFi group at week 52
Jongsma et al., 2020b [51]	2015	9.22	57	Retrospective, case–control, multicenter	2015–2019	Europe, Canada	CD, UC, IBDu	IFX		Start IFX < 10 yrs of age vs. >10 yrs	1 yr	Dosing, treatment intervals, trough levels, discontinuation, clinical remission	Equal amount of patients maintained therapy with IFX, younger patients on significantly higher dosage per kg, no effect of proactive drug monitoring
Church et al., 2019 [52]	125	14	54–70	Retrospective Single-center	2000–2015	Canada	SR UC	IFX	0.7 yrs	Standard vs. intensified induction	M = 1.4 yrs	Colectomy, remission, mucosal healing, AEs	Lower chance of colectomy in intensified regimen, other long-term outcomes are similar, 66% mucosal healing, AEs were rare
van Hoeve et al., 2019 [53]	35			retrospective	2012–2018		CD, UC	IFX		Remission at week 52 vs. non remission	52 weeks	Clinical, biological remission, trough levels	Trough levels just before maintenance were the only predictors for clinical and biological remission
Schnell et al., 2021 [54]	42	13.3, 14.27	64.3	Prospective, controlled, single-center		Germany	CD, UC	IFX		Healthy matched controls	2, 6, 12 months	Biological remission, trough levels, cytokines	Higher trough levels in patients responding to treatment after 6 months, no effect of comedication with azathioprine Before treatment different cytokine profiles in IBD patients and healthy controls
Cheifetz et al., 2022 [55]	103			Post hoc REACH trial			CD	IFX			10, 30, 54 weeks	Clinical remission	Higher infliximab concentration at week 10 was associated with clinical remission at week 10, and 30
Lawrence et al., 2022 [56]	140	14,1	54%	trial	2016–2018	Canada, Scotland		IFX		Standard induction vs. Optimization-based induction	52 weeks	Clinical remission	Higher rates of clinical remission in optimized induction
Chung et al., 2022 [57]	85			Single-center retrospective			CD	IFX				Pharmacokinetic model of infliximab clearance, clinical remission	CRP and Albumin predict trough levels, induction trough levels predict remission
Kwon et al., 2022 [58]	30	13.7	80	Prospective	2020–2021	Korea	CD	IFX				Cytokines, trough levels, clinical and biochemical remission	Higher cytokine profiles in patients not achieving remission than in patients in remission, Cut-off for higher IFX doses TNFi concentration > 27.6 pg/ml
Constant et al., 2021 [59]	55	13.1	69	Retrospective single-center	2013–2019	USA	CD	IFX			2, 8 weeks	Laboratory markers, IFX trough levels	Baseline laboratory markers (CRP, hypoalbuminemia, ESR) significantly associated with inadequate post-induction IFX trough concentration
Merras-Salmio et al., 2017 [60]	146	14.8	57	Retrospective, Single-center	2003–2014	Norway	CD, UC, IBDu	IFX	1.8			IFX trough levels, IFX ADAs	63% of patients had loss of response, trough level significantly higher in patients in remission or ongoing therapy
Dave et al., 2021 [61]	30	14.3–33.5	60	Part prospective, part retrospective	2017–2019	India	CD, UC	IFX	5			IFX trough level, ADAs, evaluation of iDose software	iDose predicted 70% of patients’ trough concentrations correctly Of 11 patients managed with iDose, 8 achieved clinical remission, 2 showed partial response, one developed antibody
Curci et al., 2021 [62]	76	14.7	47.4	Prospective, two centers		Italy	CD, UC	IFX			8, 22, 52 weeks	Clinical response	single-nucleotide polymorphisms (SNPs) rs396991 in FCGR3A variant had significantly lower trough levels, higher chance of ADAs and reduced clinical response
Clarkston et al., 2019 [63]	72	13.6	65	Prospective cohort, Single-center	2014–2018	US	CD	IFX	51 days		1 yr	Clinical response (wPCDAI), biological response, maintenance concentrations	Clinical response 64%, fecal calprotectin improvement in 54%
El-Matary et al., 2019 [64]	52	13.5	60.8	Cohort, multicenter	2014–2017	Canada	fCD	IFX			24 weeks	Fistula healing, trough levels	Correlation between pre-fourth infusion trough levels and fistula healing
Stein et al., 2016 [65]	77	14.8	63	Prospective single-center	2006–2011	US	CD	IFX	1.66 yrs		1 yr	Ongoing treatment with IFX CRP, ADAs, trough levels	78% remained on IFX associated higher week 10 trough levels
Drobne et al., 2018 [66]	183	15.4–40	57	Cohort, single-center	2010–2015	Slovenia	CD, UC, IBDu	IFX	7.3, 5.7			Trough level, CRP, fecal calprotectin	Higher trough levels were associated with lower levels of CRP and fecal calprotectin, no higher number of infections in higher trough levels
Courbette et al., 2020 [67]	111	11.6	59	Retrospective single-center	2002–2014	France	CD	IFX			14 weeks	Clinical response, predictors for response, through levels	38.7% in clinical remission plus 36% partial response Normal growth and normal albumin levels at first application associated with clinical response
Crombé et al., 2011 [68]	120	14.5	45	Retrospective registry	1988–2004	France	CD	IFX	41 months			Short- and long-term efficacy, rate of resection surgery, AEs	58% response rate, reduced risk for surgery in responder group, 13% of AEs that led to discontinuation
**Adalimumab**													
Cozijnsen et al., 2015 [5]	53	11	49.1	Observational cohort		Netherlands	CD	ADL	3 yrs		MD = 12 months	Categorized cPCDAI, discontinuation/treatment failure	64% remission after three months, maintained by 50% for two yrs, more IFX primary non-responders failed ADL than Patients with loss of response
Croft et al., 2021 [24]	93			Double blind nulticenter	2014–2018	10 countries	UC	ADL		High dose induction vs. standard dose	8, 52 weeks	Clinical remission, mucosal healing, AEs	Remission rates in ADL group better than in placebo groups, high dose induction had higher rate of remission in week 8 and week 52
Assa et al., 2019 [69]	78	14.3	71	Randomized controlled trial	2015–2018	Israel	CD	ADL		Proactive vs. reactive drug monitoring	8–72 weeks	Steroid-free remission, biologic remission, discontinuation	Significantly higher proportion of patients achieved steroid-free remission in the proactive group than in the reactive group (82% vs. 48%), as well as drug intensification (87% vs. 60%)
Matar et al., 2020 [70]	78	14.3	71	Randomized controlled trial multicenter	2015–2018	Israel	CD	ADL		With and without immunomodulator (IM)		Sustained steroid-free remission, laboratory markers, trough levels, ADAs, AEs	No difference in steroid-free remission between groups with and without IM (73% vs. 63%), or laboratory markers, trough levels, ADAs, occurrence of AEs
Dubinsky et al., 2016 [71]	188		51, 57	Randomized controlled trial multicenter		8 countries	CD	ADL	3 yrs	High dose, low dose weekly	4, 26, 52 weeks	Remission, response rate, AEs	Significantly higher proportion of patients in high dose group responded (31.4% vs. 18.8%) and achieved remission (57.1% vs. 47.9%), same rate of AEs
Payen et al., 2023 [72]	120				2008–2019		CD	ADL		Top-down vs. step-up	12, 24 months	Steroid -, EEN-free remission, clinical remission	Top-down strategy more effective, higher serum levels of ADL, no serious AEs
Lucafò et al., 2021 [73]	32	14.9	62.5	Retrospective cross sectional multicenter	2013–2019	Italy	CD, UC	ADL	41.73		4, 52, 82 weeks	Disease activity (PUCAI, PCDAI), trough levels	Around 50% remission rate, higher trough levels in patients with sustained clinical remission
**Golimumab**													
Hyams et al., 2017 [74]	35	15		Open-label multicenter	2014–2015	North America, Europe, Israel	UC	GOL	15 yrs		2, 4, 6 weeks	Serum concentration, clinical outcomes, AEs	60% clinical response, 34% clinical remission, and 54% mucosal healing, no safety concerns
**Vedolizumab**													
Garcia Romero et al., 2021 [7]	42	12.6	52.4	Retrospective, multicenter	2017–2019	Spain	CD UC	VEDO	2.6 yrs (CD), 4.1 yrs (UC)		14, 30, 52 weeks	Laboratory markers, activity indices, AEs	52.4% overall remission rate at week 14, more in UC, 84.5% remained remission in week 52
Atia et al., 2023 [75]	142	13.6	46%	Multicenter, prospective cohort, multicenter	2016–2022	6 countries	CD, UC, IBDu	VEDO			14 weeks	Steroid-free -/EEN-free remission	42% UC in remission under vedolizumab, 32% CD, optimal drug concentration at week 14—> 7µg/ml
Ungaro et al., 2019 [76]	22 pediatric	adult + pediatric		Cross-sectional, two centers		USA	CD, UC	VEDO				Clinical -, steroid-free -, biochemical remission, drug concentration	Vedolizumab concentration > 11.5 µg/mL was associated with steroid free and biochemical remission
Colman et al., 2023 [77]	74	16	51	Prospective observational	2014–2019	USA	CD, UC, IBDu	VEDO	33 months			Pharmacokinetic model, clinical remission, through levels	Final model includes weight, erythrocyte sedimentation rate, and hypoalbuminemia
**Ustekinumab**													
Yerushalmy-Feler et al., 2022 [8]	69	15.8		Retrospective, Multicenter		Europe	CD	UST	4.3 yrs		3 months	Clinical remission, CRP, fecal Calprotectin, endoscopic, histological healing	Reduction in inflammatory markers, 16% endoscopic, 13% histological mucosal healing
Dhaliwal et al., 2021 [9]	25	14.8	28	Prospective, multicenter	2018–2019	Canada	UC	UST	2.3 yrs		26, 52 weeks	Steroid-free remission, PUCAI, endoscopic remission, AEs	69% steroid free remission, significantly more of whom only failed TNFi treatment before (instead of TNFi and VEDO also)
Dayan et al., 2019 [10]	52	16.8	50, 20	Observational cohort	2014–2018	USA	CD, UC, IBDu	UST	4.9 yrs (CD) and 1.8 yrs (UC/IBDu)		52 weeks	Steroid-free remission, clinical and biomarker remission	75% maintained on UST after one yr, 50% of bio-exposed and 90% of bio-naïve in steroid free remission

Note: Only studies including pediatric patients receiving TNFi are included in the table. ADAs = anti-drug antibodies, ADL = adalimumab, AEs = adverse events, CD = Crohn’s disease, EEN = exclusive enteral nutrition, fCD = fistulizing Crohn’s disease, GOL = golimumab, IBDu = unclassified IBD, IFX = infliximab, IM = immunomodulator JIA = juvenile idiopathic arthritis, lCD = luminal Crohn’s disease, MINI = Mucosal Inflammation Non-invasive Index, (w)PCDAI = (weighted) pediatric Crohn’s disease activity index, PEN = partial enteral nutrition, PsA = psoriasis arthritis, PUCAI = pediatric ulcerative colitis activity index, SR UC = steroid refractory, TNFi = TNF-inhibitors, UC = ulcerative colitis, UST = ustekinumab, VEDO = vedolizumab, VEO IBD = very early onset inflammatory bowel disease, yrs = years.

## Abbreviations

ADA	Anti-drug Antibodies
BMI	Body mass index
CD CEDATA CMV	Crohn’s disease Registry of Pediatric Patients with IBD in German-speaking countries Cytomegaly virus
CRP	C-reactive Protein
EIM	Extraintestinal manifestations
ESPGHAN	European Society for Pediatric Gastroenterology Hepatology and Nutrition
GPGE	German Association of Pediatric Gastroenterology
IBD	Inflammatory bowel disease
IFX	Infliximab
PCDAI	Pediatric Crohn’s disease activity index
PUCAI UC	Pediatric ulcerative colitis activity index Ulcerative colitis
TNF	Tumor necrosis factor
TDM	Therapeutic drug monitoring
